# Influence of Nitric Oxide generated through microwave plasma on L6 skeletal muscle cell myogenesis via oxidative signaling pathways

**DOI:** 10.1038/s41598-017-00154-3

**Published:** 2017-04-03

**Authors:** Naresh Kumar, Priyanka Shaw, Han Sup Uhm, Eun Ha Choi, Pankaj Attri

**Affiliations:** Plasma Bioscience Research Center/Department of Electrical and Biological Physics, Kwangwoon University, 20 Kwangwon-Ro, Nowon-Gu, Seoul 139-701 Korea

## Abstract

Myogenic precursors are myoblasts that have a potency to differentiate into muscle fibers on injury and maintain the regenerative power of skeletal muscle. However, the roles of exogenous nitric oxide (NO) in muscle development and myoblast differentiation are largely unknown. Therefore, in this study, we examined the effects of exogenous NO generated by a microwave plasma torch on rat myoblastic L6 cell proliferation and differentiation. We observed that the differentiation of L6 myogenic precursor cells into myotubes was significantly enhanced after NO treatment. The expression of the myogenesis marker proteins and mRNA level, such as myoD, myogenin, and myosin heavy chain (MHC), as well as the cyclic guanosine monophosphate (cGMP) level, were significantly increased after the NO treatment, without creating toxicity. Moreover, we observed that the oxidative stress signaling [extracellular-signal-regulated kinase (Erks), ﻿and Adenosine monophosphate-activated protein kinase (AMPK)] phosphorylation was higher in NO treated cells than in the control cells [without NO treatment]. Therefore, these results reveal the exogenous NO role in regulating myoblast differentiation through the oxidative stress signaling pathway. Through this work, we can suggest that exogenous NO can help in cell differentiation and tissue regeneration, which provides new possibilities for plasma medicine.

## Introduction

Nitric oxide (NO) plays an important role in regulating the cellular signaling molecule involved in many physiological and pathological processes. This simple molecule has a broad scope in the biological cellular functions that balance vascular homeostasis, including immunomodulation, regulation of cell growth, differentiation, and during wound healing; it also protects the vessel from the fatal influences that effect the platelets and cells in blood circulation^1–5^. In fact, diminished nitric oxide bioactivity can cause various pathogenesis and progression of vascular disorders, hypertension, Alzheimer’s disease, hypercholesterolemia, and myocardial ischemia diseases^5–7^. Recently, George Han *et al.* developed NO-releasing nanoparticles that modulate and accelerate wound healing in a pleiotropic manner^8^. In another study, Mostafa *et al.*, demonstrated that exogenous NO generated by plasma can facilitate early osteogenic differentiation without the presence of growth factors in media. They also claimed that exogenous NO could be transported to an area of interest to activate the osteoprogenitor cell without subsequent toxicity^9^. Other research groups generated the NO using arc discharge and demonstrated that treatment with NO has the ability to heal the damaged tissue and wounds of rats and humans^10–12^. In a previous study, we developed a microwave plasma torch system to generate NO and verified the effects of NO on non-pathogenic saprophytic fungus (*Neurospora crassa*) cell differentiation^10^. The above studies suggest that exogenous NO and NO inducible agent can be used for various biomedical applications. However, the beneficial effects of NO generated through plasma have not been intensively explored for various applications.

The cell proliferation, differentiation, and regeneration of injured tissue are essential steps in wound healing. However, injured muscle tissue itself has an ability to repair and regenerate through myogenic differentiation^13–16^. Many studies have been reported in recent years on the role of non-thermal atmospheric pressure plasma on wound healing^17, 18^. Although, the plasma generated NO can possibly stimulate these steps, studies on plasma generated NO remain absent from the literature. The rat skeletal muscles (L6) cell line can reproduce myogenic differentiation in the presence of a growth factor in a culture medium and has been the most widely used model to investigate myogenic differentiation^19, 20^. However, the expression and sub cellular localization of NO in muscle development and myoblast differentiation are largely unknown. In order to extend this study, further investigation on the differentiation and regeneration in advanced cell types is needed. Therefore, in the present work, we studied the role of exogenous NO generated by a microwave plasma torch on the proliferation/differentiation of rat myoblastic L6 cells. Additionally, we studied the expression of myogenesis marker proteins and mRNA levels such as MyoD, myogenin, and myosin heavy chain (MHC), as well as phosphorylation of extracellular-signal-regulated kinase (Erks), adenosine monophosphate-activated protein kinase (AMPK), and cyclic guanosine monophosphate (cGMP) levels before and after the exogenous NO exposure. In addition, we attempt to clarify the molecular mechanism and role of exogenous NO produced by a microwave plasma torch in myogenic differentiation.

## Results

### Variation in physical and chemical parameters

**Figure 1:**
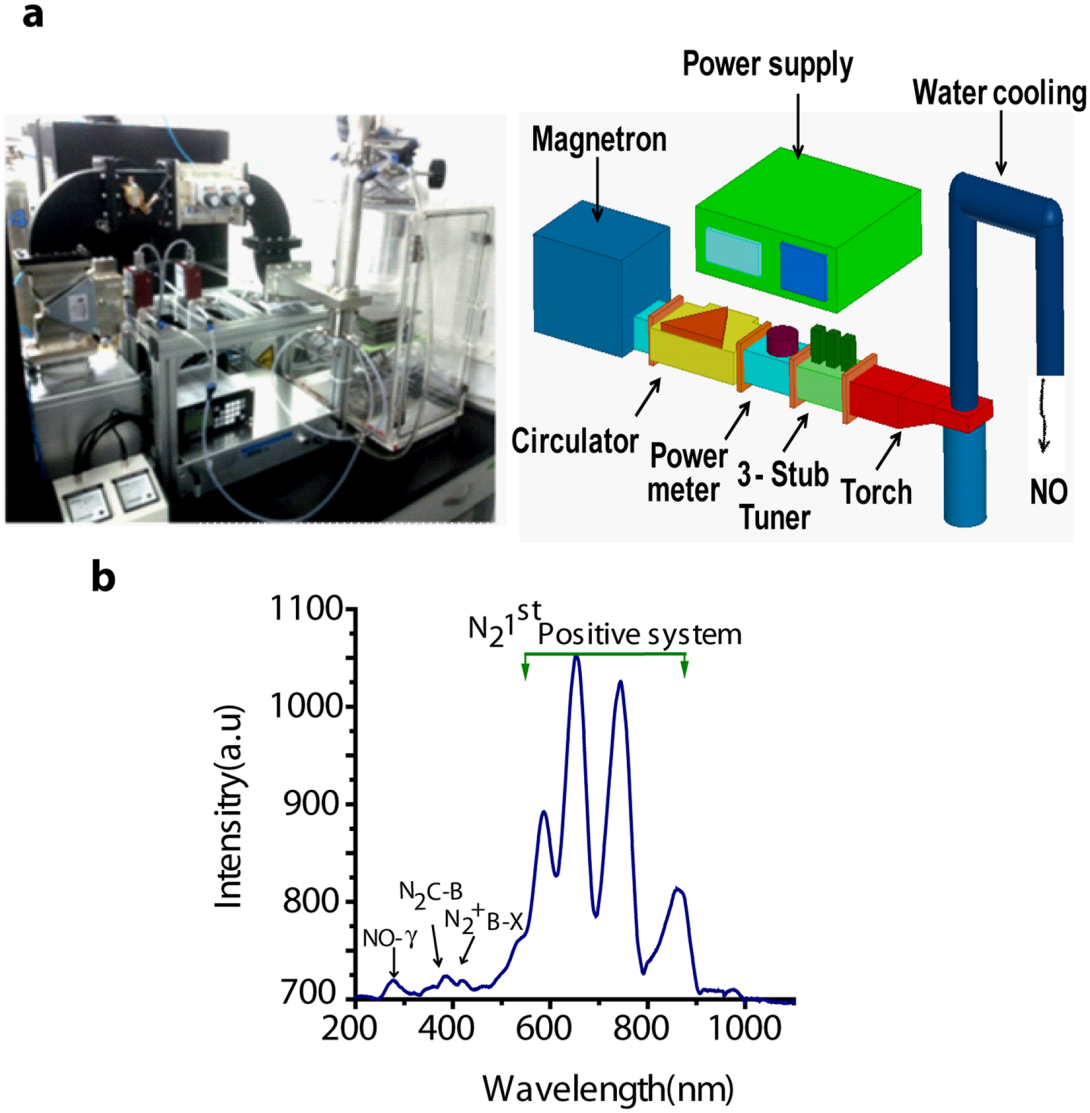
(**a**) Symmetrical diagram of Microwave plasma torch, (**b**) OES spectra of NO production using microwave plasma torch.

The production of NO was investigated using N_2_ (nitrogen) plasma operated at a microwave power of 400 W with an N_2_ flow rate of 10 lpm (liters per minute). Different (200~400) standard cubic centimeters per minute (SCCM) of O_2_ (oxygen) gas were applied and the various excited plasma species were identified using optical emission spectroscopy (OES) with a wide wavelength range of 200–900 nm, as shown in Fig. 1b. The emission spectra show the presence of excited N_2_ species, whereby the first positive system of N_2_ is produced due to the molecular excitation of N_2_. In addition, Fig. 1b shows the highly reactive NO radicals at 250 nm, whereas the atomic oxygen lines at 616 and 777.1 nm are immersed due to the presence of the N_2_ first positive system strong emission lines. The N_2_ first positive system was related to the excited metastable state of N_2_ molecules due to the small mole fraction of the O_2_ gas. After applying 10 lpm of N_2_ and a certain amount of O_2_ in a microwave plasma torch, mostly a gaseous form of NO was produced.

### Change in physical and chemical properties of culture media after exogenous NO exposure

**Figure 2:**
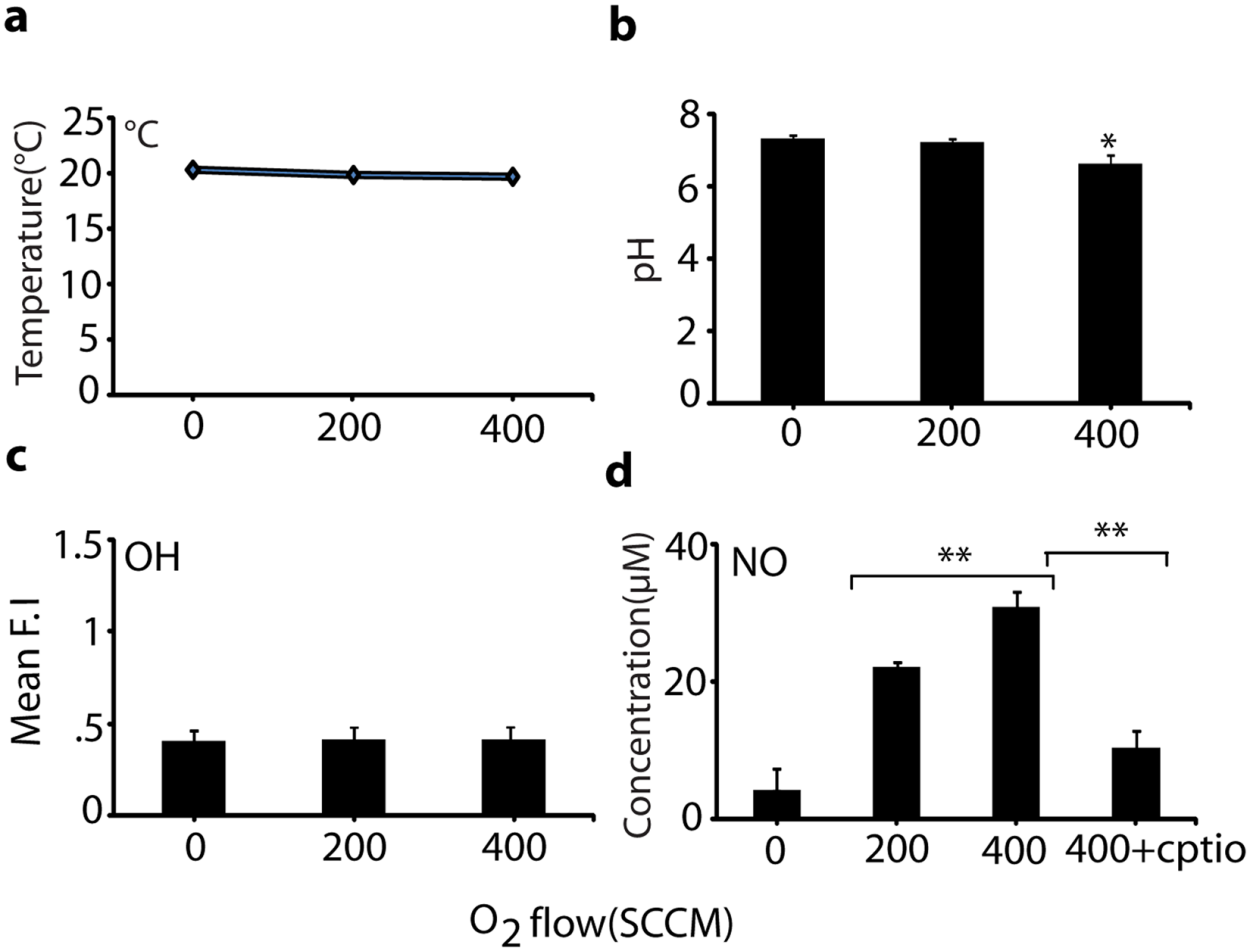
Temperature, pH, and ROS/RNS measurements after NO exposure in media. (**a**) Measurement of Temperature, (**b**) pH, (**c**) Determination of OH radicals and (**d**) Determination of NO concentration in 2 ml media after NO (200 and 400 SCCM of O_2_ flow rate) for 3 min. All values are expressed as ±SD in triplicate. Students t-test was performed as the control (* denotes P < 0.05 and ** denotes P < 0.01).

In order to identify the amount of NO responsible for cell differentiation, we applied O_2_ flow rates of 200 and 400 SCCM and 10 lpm of nitrogen in a microwave plasma torch to generate NO. The process was applied to a culture media for further analysis of NO concentration (using a detection kit, as given in detail in the Materials and Methods section). We checked the temperature and pH of the culture media after NO (O_2_ flow rates of 200 and 400 SCCM) for 3 min exposure; no significant change in temperature was observed (Fig. 2a). As shown in Fig. 2a and b, the temperature of the media was maintained at around 20 °C during NO treatment, but the pH of the media slightly decreased after NO treatment with an O_2_ of 400 SCCM. These data indicate that pH and temperature had no effect on cellular differentiation at an O_2_ flow of 200 SCCM. We also checked the amount of OH radicals in the DMEM media after the NO exposure and found that the fluorescence intensity of the hydroxyterephthalic acid (HTA) formation had not changed significantly with the variation of O_2_ flow of 200 SCCM and 400 SCCM compared with the control, as shown in Fig. 2c. This reveals that there is either no production, or a very small production, of OH radicals in the NO treatment, so the effect of OH radicals was negligible in our study. We also found that NO concentration in DMEM media for O_2_ supply of 200 and 400 SCCM for 3 min was ~25 and ~35 µM, respectively (Fig. 2d). However, in the presence of the NO scavenger [2–4-carboxyphenyl-4, 4, 5, 5- tetramethylimidazoline-1-oxyl-3-oxide (cPTIO)] at an O_2_ flow of 400 SCCM, the concentration of NO in media decreased, demonstrating that the exogenous NO value can be controlled in media using a scavenger. Further, we analyzed the NO effects generated by the microwave plasma torch on cell viability, proliferation, and toxicity to confirm whether the NO treatment triggered the elevation of cell differentiation.

### Toxicity test at different O_2_ flow rates

**Figure 3:**
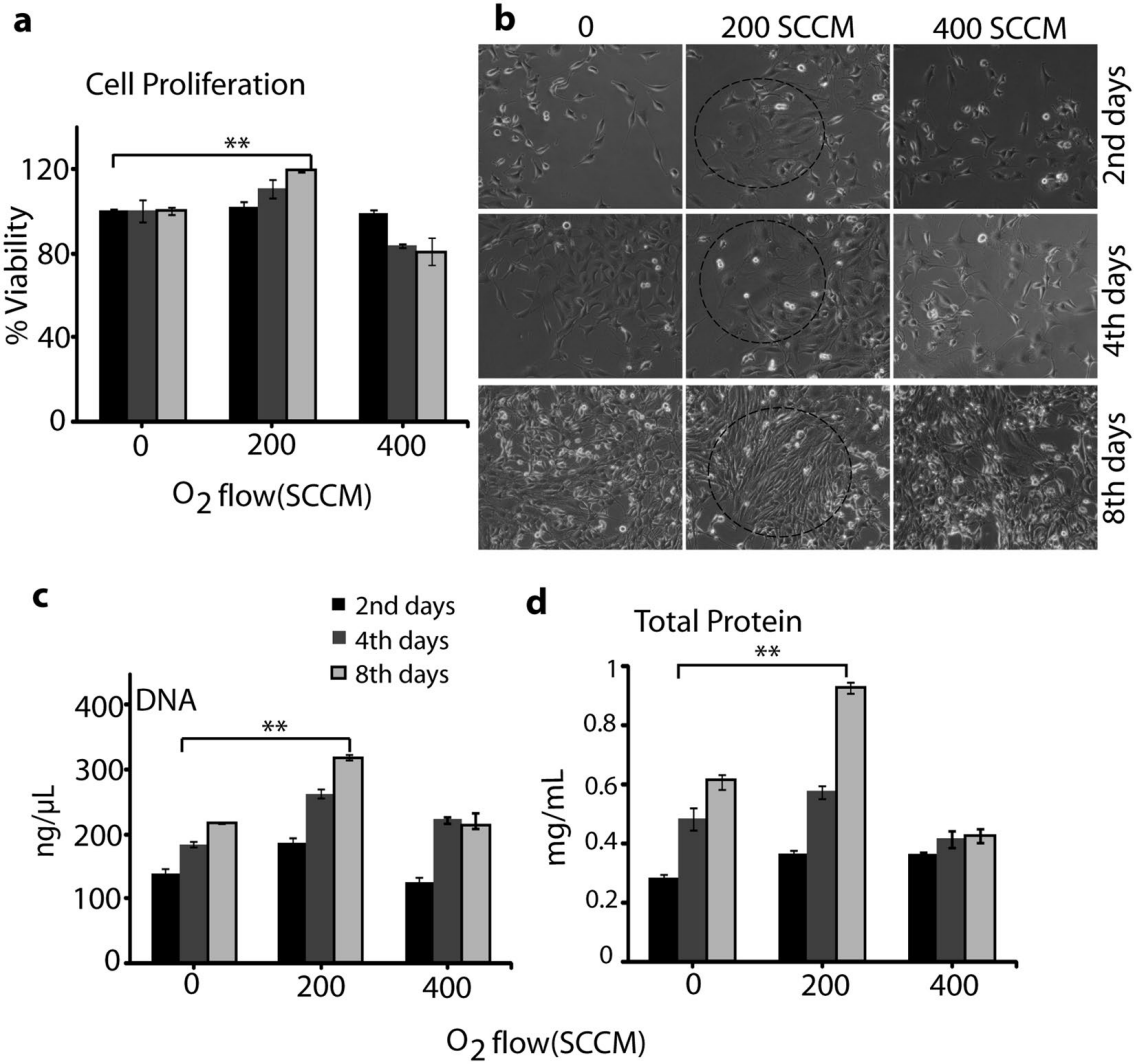
L6 muscle cells proliferation, differentiation, DNA, and total protein content analysis after NO exposure. (**a**) Cell proliferation analysis, (**b**) Cell differentiation analysis using light microscopy, (**c**) DNA estimation, and (**d**) Total protein measurement after exposure with NO (200 and 400 SCCM of O_2_ flow rate) for 3 min day 2, day 4, and day 8. All values are expressed as ±SD in triplicate. Students t-test was performed as the control (* denotes P < 0.05 and ** denotes P < 0.01).

To determine the cell viability and toxicity of NO on L6 cells, we performed an MTT [3-(4,5-Dimethylthiazol-2-yl)-2,5-Diphenyltetrazolium Bromide] assay. After treating with NO at an O_2_ flow of 200 SCCM for 3 min, the L6 cells showed significant proliferation on the day 2, day 4, and day 8. However, at an exposure of 400 SCCM O_2_, toxicity in the L6 cells was observed (Fig. 3a). The light microscope image in Fig. 3b shows that the cellular differentiation after exposure with NO (O_2_ flow of 200 and 400 SCCM) for 3 min on the day 2, day 4, and day 8. This shows that the 200 flow of O_2_ significantly affected the cellular differentiation on every consecutive day, but the O_2_ flow of 400 showed toxicity. The total DNA and total protein contents of cells after treating with O_2_ flow of 200 SCCM was greater than that of the cells treated with O_2_ flow of 400 SCCM (Fig. 3c and d). For example, the DNA and protein contents at treatments of O_2_ of 200 SCCM on the day 2, day 4, and day 8 of culture was significantly higher. However, the total DNA and total protein contents reduced at an exposure of O_2_ flow of 400 SCCM. Under the exposure of O_2_ flow of 200 SCCM, confluent myoblasts cell layers were observed after 2 days of culture cell proliferation, which continued to increase on the 4^th^ and 8^th^ days of culture. Hence, the total DNA and total protein contents indicate that the exposure of NO with an O_2_ flow of 200 SCCM induces cell proliferation. Hereafter, we used NO generated due to O_2_ flow of 200 SCCM as the NO for the remains of the study.

### Cell morphology and differentiation study

**Figure 4:**
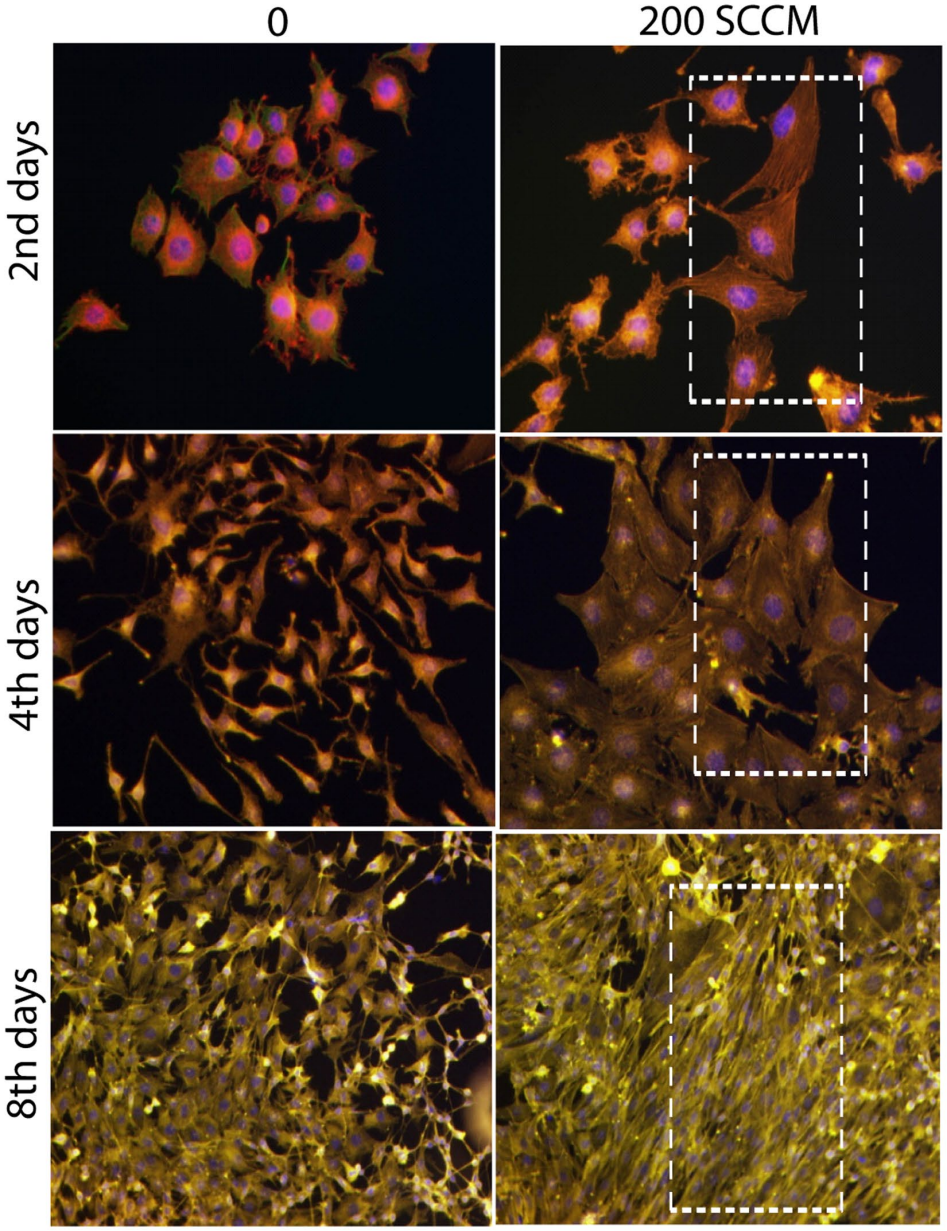
Cellular differentiation analysis through actin, tublin and nuclear immunofluorescence images of L6 cells on day 2, day 4, and day 8 after exposure with NO (200 and 400 SCCM of O_2_ flow rate) for 3 min.

For a clearer observation of the myoblast differentiation after NO exposure, we performed immunostaining of actin and α-tubulin filament on the day 2, day 4, and day 8 of incubation. The average area of the individual myotube was stained with monoclonal antibodies of α-tubulin conjugated with fluorescein isothiocyanate (FITC) (Abcam, USA) and the actin filament was stained with rhodamine phalloidin (Molecular probe, USA). During the extended cell culture, the L6 myoblast showed a highly organized structure after differentiation into myotubes (Fig. 4, with separate actin/tublin image shown in Supplementary Figures S1–S3). In particular, the majority of the cells cultured on the 4^th^ day of incubation and the 8^th^ day incubation showed that the differentiated myotubes were longer and wider than those observed at the 4^th^ and 8^th^ days without exposure.

### Myogenesis related gene and Myosin heavy chain protein expression analysis

**Figure 5:**
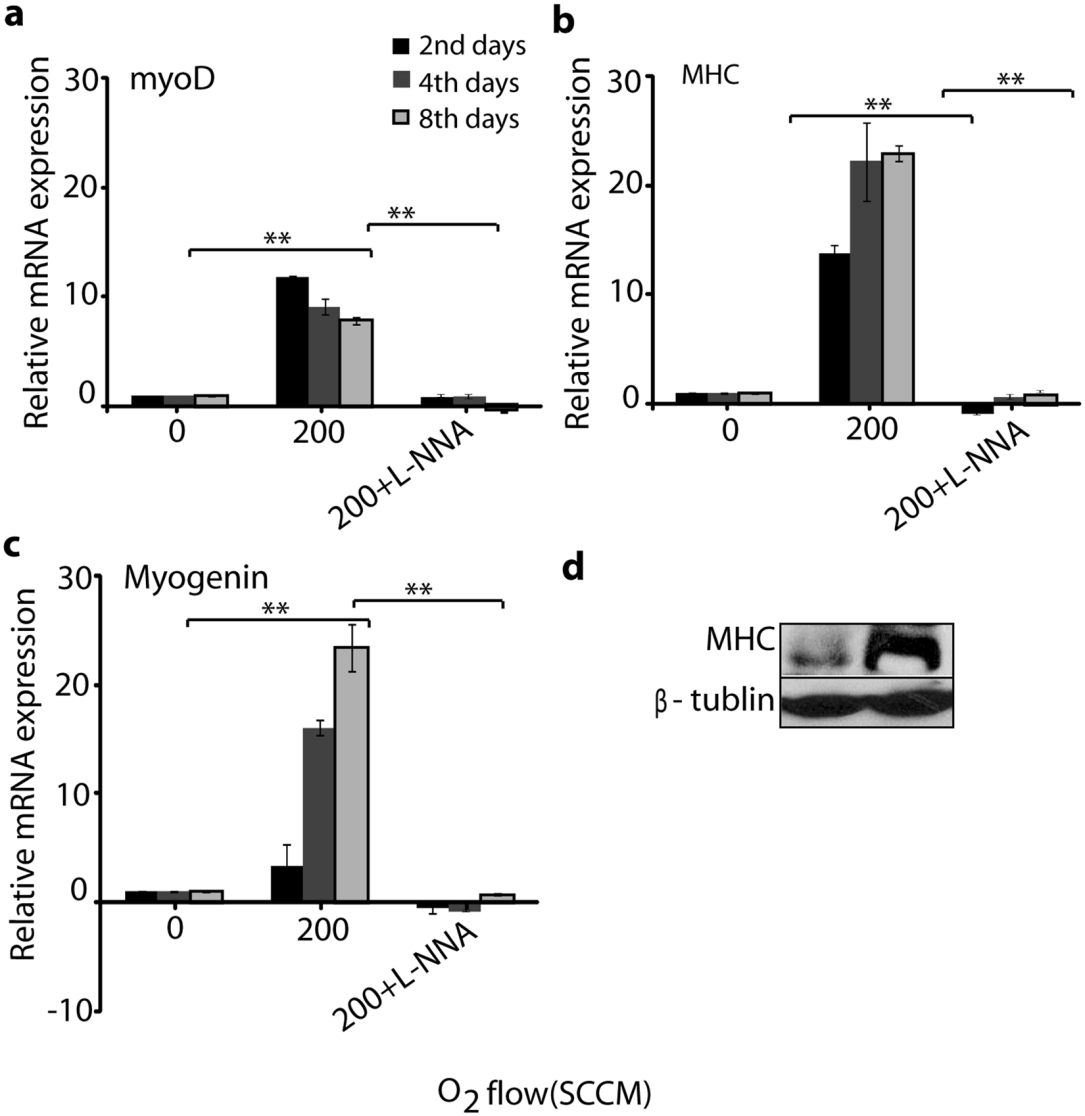
Myogenic differentiation regulated mRNA expression and myosin protein expression analysis after exposure with NO. (**a**) myoD, (**b**) MHC, and (**c**) myogenin gene expression analysis after exposure with NO (200 of O_2_ flow rate) for 3 min with and without the presence of L-NNA on day 2, day 4, and day 8. The relative value of mRNA expression of these genes were measured by real-time RT-PCR. β-actin was used as a reference gene. All values are expressed as ±SD in triplicate. Students t-test was performed as the control (* denotes P < 0.05 and ** denotes P < 0.01). (**d**) Western blot analysis of the myosin on 8^th^ day after exposure with NO (200 of O_2_ flow rate) for 3 min. β-tublin was used as the loading control.

To complement the cellular differentiation analysis, we investigated the expression of myogenic differentiation markers after exogenous NO exposure. We found that the myogenin, myoD, and MHC genes were significantly over expressed in cells exposed with NO compared to the control, while the myoD gene was expressed early in all groups. A significant difference was shown in the expression level of the myoD gene on the 2^nd^ day compared with that on the 4^th^ and 8^th^ day of incubation among the NO treated groups, as shown in Fig. 5a. However, other genes such as MHC and myogenin, were expressed during myogenic development at the early, late, and fully differentiated stages. For example, the level of MHC and myogenin gene expression (Fig. 5b and c) was higher on the 4^th^ day and 8^th^ day of incubation, compared with the control. However, in all treated groups, MHC protein expression showed higher expression on the 8^th^ day of incubation (Fig. 5d with separate whole blot image in Supplementary Figure S5). These results indicate that NO generated by microwave plasma torch influences myoblast differentiation. However, we also studied the gene expression of myoD, myogenin, and MHC in the presence of N^G^-nitro-L-arginine (L-NNA). The expression of these genes were down-regulated, indicating that L-NNA inhibits the NO synthase to control the intracellular NO production^21, 22^. Therefore, the above results provide strong evidence that extracellular NO generated by a microwave plasma torch influences the myoD, myogenin, and MHC gene expression.

### Intracellular Nitric oxide and oxidative stress marker analysis

**Figure 6:**
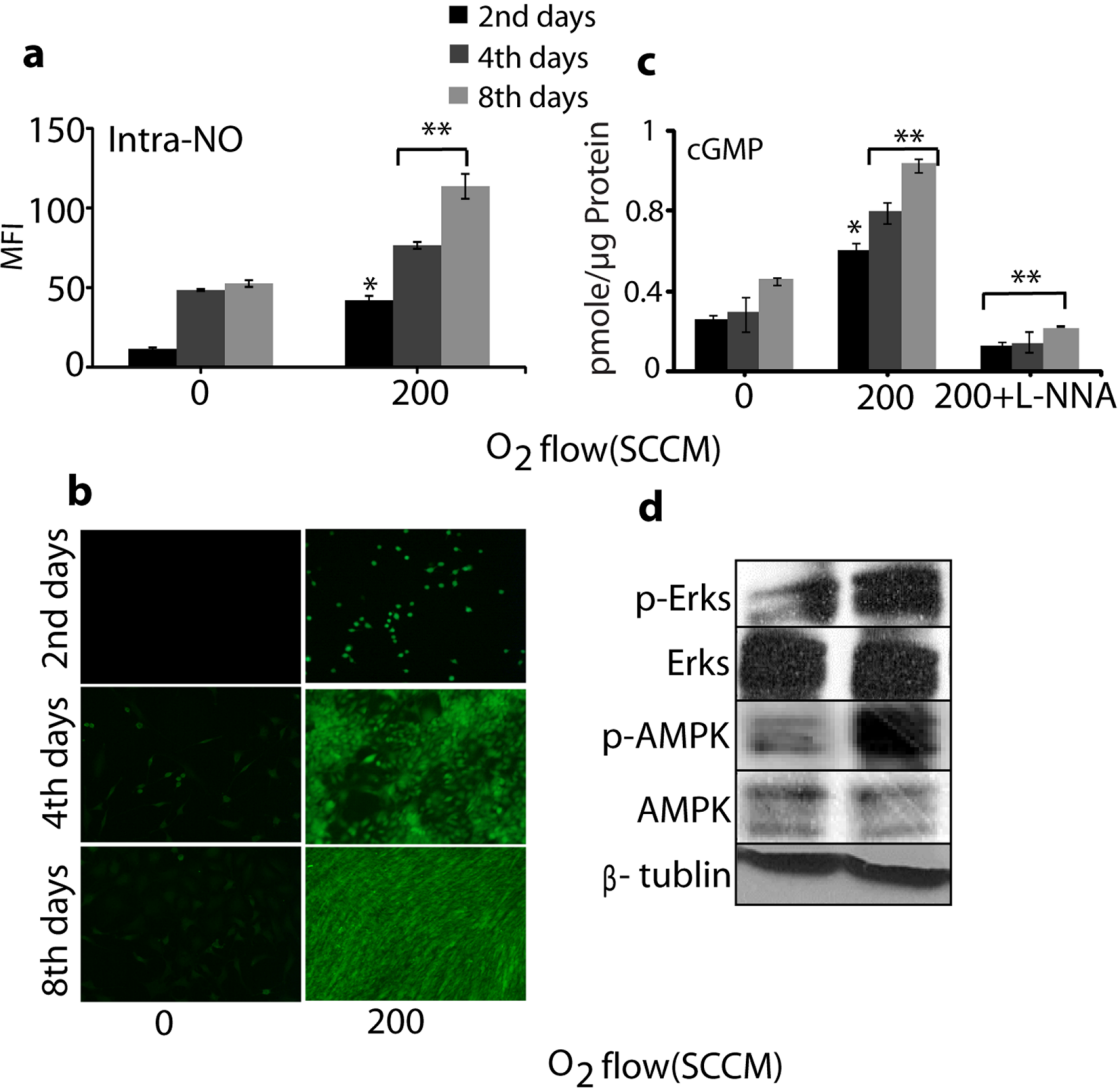
Intracellular NO and oxidative markers analysis. (**a**) Level of intracellular NO, (**b**) Fluorescence image of intracellular NO (200 of O_2_ flow rate) for 3 min with on day 2, day 4, and day 8 (**c**) cGMP analysis after exposure with NO (200 of O_2_ flow rate) for 3 min with and without the presence of L-NNA on day 2, day 4, and day 8. Each value is the average of three technical replicates. Students t-test was performed as the control (* denotes P < 0.05 and ** denotes P < 0.01), (**d**) Western blot analysis of the Erks and APMK phosphorylation on 8^th^ day after exposure with NO (200 of O_2_ flow rate) for 3 min. β-tublin was used as the loading control.

The DAF-FM diacetate fluorescent probe was used to detect the total intracellular NO level, which significantly increased for 3 min exogenous NO treatment in comparison with the control without exposure on the day 2, day 4, and day 8, as shown in Fig. 6a and b. Additionally, we studied the change in concentration of cGMP after the treatment with exogenous NO. The increase in the cGMP level was activated by NO, which solubilized the guanylate cyclise by binding to the enzyme’s heme moiety and subsequently increased the cGMP level. From Fig. 6c, we observed an increase in the pmole/µg protein in the cGMP formation. The maximum cGMP was formed after 3 min exposure of exogenous NO. On the other hand, the concentration of cGMP decreased after treatment of cells in the presence of L-NNA and exogenous NO for 2 days, 4 days, and 8 days. Therefore, the presence of L-NNA demonstrates the enhancement of intracellular NO is generated through extracellular NO, using a microwave plasma torch. This Exogenous NO in media were responsible for the oxidative stress inside the cell and also modulated several types of protein signaling such as extracellular signal–regulated kinases 1/2 (Erk1/2) and adenosine monophosphate-activated protein kinase (AMPK), which were involved in the regulation of cell survival, tissue regeneration, and oxidative stress^23, 24^. Thereafter, we checked the Erks and AMPK phosphorylation on the 8^th^ day immediately after 3 min treatment of exogenous NO. Figure 6d (separate whole blot image in Supplementary Figure S5) shows that for 3 min exogenous NO treatment, a greater phosphorylation was observed, while the expression of these proteins did not show significant change. Collectively, our findings show that NO generated using a microwave plasma torch might be used to activate differentiation and myotube formation in myoblasts. This reveals the importance of exogenous NO presence in media on the activation of a signaling target for skeletal muscle regeneration.

## Discussion

**Figure 7:**
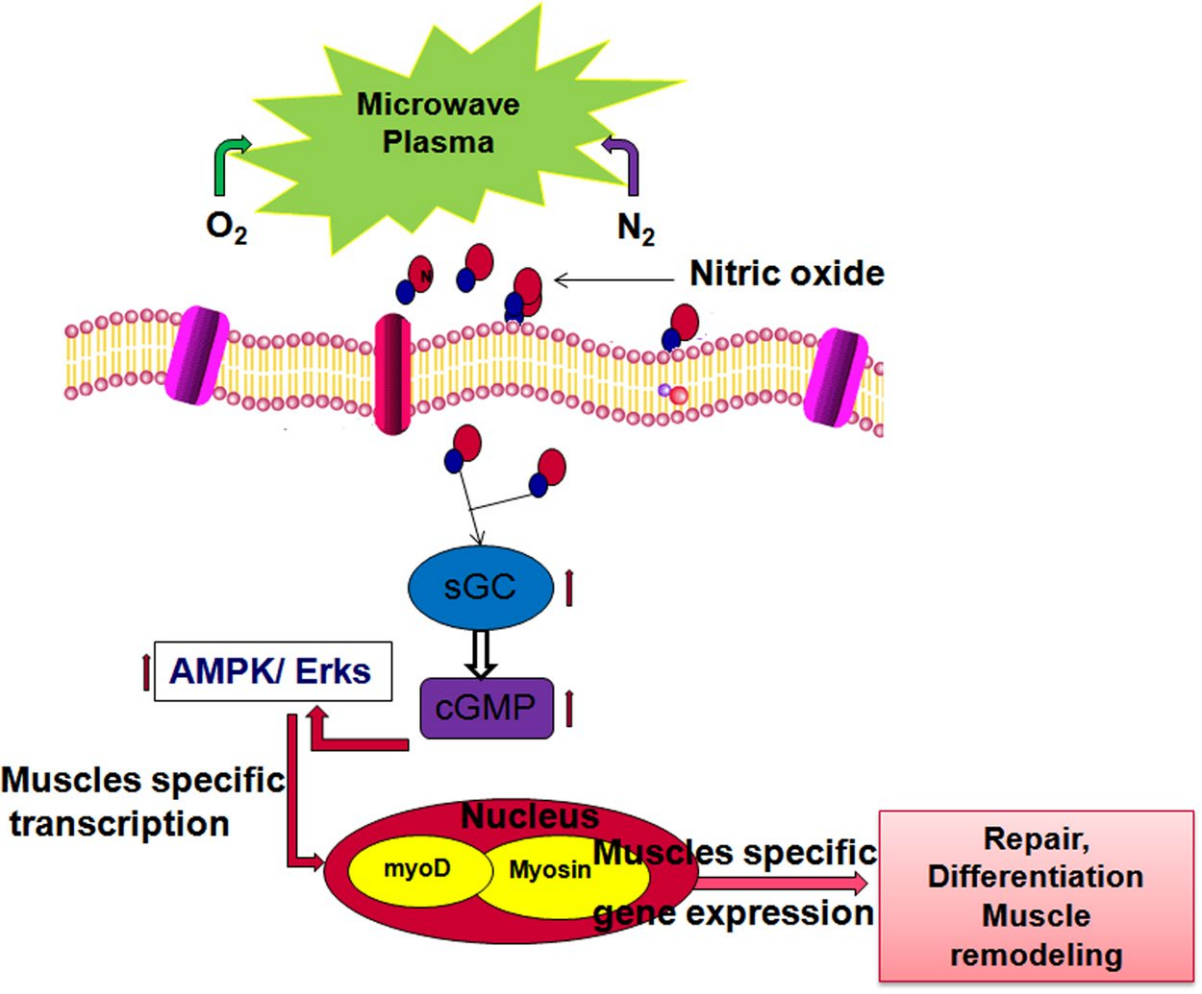
Nitric Oxide generated through microwave plasma penetrates inside the cell through the cell membrane that activates the myogenic specific gene as well as protein via a oxidative related signaling pathway, which leads to cell proliferation and differentiation in L6 skeletal muscle cells.

In continuous studies of plasma biological applications, we developed a microwave plasma torch that can generate NO species^10^. In addition, to understand the effect of NO molecular action on cell differentiation, we investigated the role of exogenous NO on the proliferation and differentiation of L6 muscle cells. Analysis of the mechanical properties of the NO demonstrated that as the O_2_ flow rate was increased, the NO generation increased. The production of NO was investigated using N_2_ plasma operated at a microwave power of 400 W with an N_2_ flow rate of 10 lpm and application of two different flow rates of O_2_ (200 and 400 SCCM). First, we determined the pH and temperature of the media after 3 min exposure to O_2_ of 200 and 400 SCCM. No significant change was observed in the pH and temperature of the culture media at an O_2_ flow of 200 SCCM. However, the NO level in the DMEM media was higher after exposure of 400 SCCM O_2_ flow, compared with the 200 SCCM O_2_ flow. Although no significant OH species was found in the media during exposure, this result indicates that the NO species have the most influence on the cells differentiation. In our previous reported work, the 3 min exposure of O_2_ at 400 SCCM showed a larger amount of NO_3_^−^ ions that might be responsible for the decrease of the pH values^10^. Further, we analyzed the NO dose generated using the microwave plasma torch on cell viability, proliferation, and toxicity to confirm that the NO treatment may have triggered the elevation of cell differentiation. Skeletal muscle tissue regeneration depends on the reoriented properties of myoblast and their potential to proliferate and differentiate to form organized muscle fibers^19, 23^. In this study, we found that the NO treatment for 3 min at an O_2_ flow of 200 SCCM has significant effects on cell proliferation and differentiation. After morphological analysis, we investigated the exogenous NO effect on the expression of myogenic differentiation markers. We found that exogenous NO exposure for 3 min significantly elevates the expression of myogenic precursor genes such as myoD, MHC, and myogenin, which indicates the formation of new muscle tissue^25–27^. NO generated through the use of a microwave plasma torch freely diffuses through the cell membranes to reach its targets at different intracellular locations and activates the signaling cascade^25^, as shown in Fig. 7. We also observed a decrease in the expression of the myoD, MHC, and myogenin genes expression in the presence of L-NNA. This demonstrates that the presence on L-NNA inhibits the NO synthase^21, 22^, only to control the intracellular NO production. Although the extracellular NO influences the myoD, the MHC as well as the myogenin expression might be possible through the activation of intracellular NO. Simultaneously to conformation this result we study the action of nitric oxide donor S-nitroso-L-glutathione (GSNO) on NOS activity and myoD, MHC, and myogenin gene expression. In this results (Figure S4a,b) we found that 200 SCCM of O_2_ and GSNO have similar effects on NOS activity as well as myogenesis related gene expression. Our results indicate that the expression of myoD, MHC, and myogenin is significantly influenced by the extracellular NO in media which is generated by the microwave plasma torch. On the other hand nitric oxide donor also influence the myoD, myogenin and MHC gene expression might be responsible for NOS activity. NO can provoke long lasting changes in the cell and signaling pathways that regulate the L6 skeletal muscle differentiation; this function of NO is manifested in its ability to regulate gene and protein expression^14^. AMPK is a serine/threonine kinase that works as a stimulant of the cellular energy that is responsible for cellular stresses. More recent studies show that AMPK also has a wider role in cellular processes such as cell proliferation, angiogenesis, and inflammation^23^. On the other hand, Erks are important signaling nodes in the pathways that control metabolism, growth, and expression^28–32^. Erks influences the signaling pathways^33, 34^ that involve eNOS; good evidence for the direct phosphorylation of eNOS by Erks can be seen in bovine aortic endothelial cells (BAECs)^35^. The increased cellular cGMP also involved in the activation promotes the phosphorylation of the Erks as well as other protein signaling^36, 37^.

In addition NO in skeletal muscle regulates metabolism related signaling, as well as demand of energy^38, 39^. that’s contribute mitocondrial biogenesis, imporoved motor function, physiological function with aging^40–42^ and contributes to muscle repair following acute and chronic muscle injuries^43–45^. Several groups also have explored the mechanisms beyond the therapeutic potential of NO and found that it has multiple actions on survival, activation and differentiation of mononuclear myogenic precursor cells. This action inhance the ability of these cells to repair the damaged muscle^46–48^. Therefore to reveal the targets of NO in the cell, our results show that 3 min NO exposure increases the expression of Erks and AMPK phosphorylation, while increasing the cGMP level. This demonstrates that exogenous NO can influence the signaling pathways in cells. To the best of our knowledge, our study provides evidence for the first time that the differentiation of myoblast can be modulated by extracellular NO produced using a microwave plasma torch. Moreover, our experimental results establish the role of exogenous NO in media and cGMP/Erks/AMPK in regulating myoblast differentiation and explain their mechanism of action, providing a direct link with oxidative stress signaling, which is a key player in myogenesis. Based on these findings, the understanding of how NO is generated by plasma offers new possibilities for plasma medicine has increased.

## Materials and Methods

### Materials

Cell culture reagents including fetal bovine serum (FBS), Dulbecco’s modified eagle’s medium including high glucose (DMEM), Dulbecco’s phosphate buffered saline (PBS), trypsin/EDTA, and penicillin–streptomycin were purchased from Gibco BRL (Carlsbad, CA, USA). Genomic DNA extraction and RNA extraction were performed using a DNA extraction kit (Gene All, CELL SV MAXI, BanseokBld, Seoul, Korea) and RNA extraction kit (rneasy mini kit, qiagen). cGMP was measured using an ELISA kit (Abcam. USA). DAF-FM Diacetate (4-Amino-5-Methylamino-2′,7′-Difluorofluorescein Diacetate, Invitrogen, USA) was used for the detection of intracellular NO. NO scavenger 2- to 4-carboxyphenyl-4,4,5,5-tetramethylimidazoline-1-oxyl-3-oxide (cPTIO) were purchased from Sigma Aldrich, USA.

### System configuration of nitric oxide generating microwave plasma

The microwave plasma system for NO generation consists of a magnetron, waveguide component (WR-340 for 2.45 GHz) and a microwave plasma torch, as shown in Fig. 1. N_2_ gas mixed with O_2_ enters the discharge tube through feeders as a swirl gas that creates a vortex flow in the discharge tube, where a plasma torch at a temperature of 6000 K and a plasma density in the order of 1013 cm^−3^ is generated. The design and operation of the atmospheric microwave plasma torch are reported in detail in the literature^10, 49^. The gas emitted from the torch flame has a very high temperature. Therefore, the microwave plasma-torch system is connected to a water cooling system for possible medical applications. The gas is monitored by a gas analyzer. The N_2_ torch flames were at a microwave power of 400 W and the N_2_ working gas is mixed with O_2_ with a small mole fraction of 10 lpm of N_2_ gas with an oxygen mixture gas of 200 and 400 SCCM.

### pH and temperature change in culture media

DMEM media in a 6-well plate (2 ml per well) were exposed for 0 and 3 min at 200 and 400 SCCM O_2_ flow rates, respectively. After exposure, the pH and temperature of the media were measured using a pH meter (Eutech Instruments, Singapore) and Infrared (IR) camera (Fluke Ti100 Series Thermal Imaging Cameras, UK).

### Culture of mouse skeletal muscle cells (L6)

L6 myoblasts were used to study the cell proliferation and differentiation in 6-well plates after exposure with NO. Cells were cultured in DMEM and supplemented with 10% FBS and 1% penicillin–streptomycin (p/s) under standard culture conditions (37 °C, 5% CO_2_). Cells were seeded onto the 6-well plate at a density of 5 × 10^4^ cell/mL and cultured under growth media for 24 h. To induce myotube formation, the media were then replaced with differentiation media (DMEM supplemented with 1% horse serum and 1% p/s) and cultured for an additional 8 days. The cells were exposed for 0 and 3 min at an O_2_ flow rate of 200 and 400 SCCM, respectively. Untreated media were included as a 0 min treatment in each experiment. After exposure for 2 days, 4 days, and 8 days incubation, the treated L6 cells were used to check the viability through MTT (3-[4,5-dimethylthiazol-2yl]-2,5-diphenyltetrazolium bromide) assay^50^.

### Measurement of DNA and protein content

To measure the DNA content at the designated time points (at the day 2, day 4, and day 8), the cells seeded in the 6-well plate were measured using a DNA assay kit. All of the cell protein extracts from the treated/untreated cells were lysed in a (RIPA) buffer (Cell Signaling Technology, USA) and the extracted protein and protein concentration were measured using the protein assay kit (Biorad, USA).

### Immunofluorescence staining

After exposure for 2 days, 4 days, and 8 days incubation, the treated muscle cells were used to check the cell morphology, and the cells on the cover glass were immunofluorescence stained for α-tublin and actin which were visualized using a fluorescence microscope (TE-2000, Nikon Corp., Tokyo, Japan). The seeded cells were fixed in 4% paraformaldehyde (in PBS) and then permeabilized in cytoskeleton buffer (pH 6.8, 50 mM NaCl, 150 Mm sucrose, 3 mM MgCl_2_, 50 mM Trizma-base, 0.5% Triton X-100). After permeabilization, non-specific binding sites were blocked by incubation with 5% FBS in PBS, and then sequentially incubated with FITC-conjugated α-tublin (1:50), and the Cell nuclei and actin were stained with DAPI (1:5000) and Rhodamine-phlloidine (1:200).

### Real-time RT-PCR

Real-time (RT-PCR) was used to measure the expression levels of myoD, myogenin, and MHC. The total RNA from the cells were cultured after exposure for 3 min at an O_2_ flow of 200 SCCM and 200 SCCM + 3 min exposure in the presence of 50 µM L-NNA (NOS inhibitor) on the day 2, day 4, and day 8, incubation. A standard 50 µM nitric oxide donor GSNO was used separately to check the expression levels of myoD, myogenin and MHC on the 8 day incubation. The total RNA was isolated and the concentration, purity, and integrity of the isolated RNA were assessed using a UV-Vis spectrophotometer (Beckman, Fullerton, CA, USA). cDNA synthesis was performed on RNase-free DNase-treated total RNA (1 mg) using a reverse transcription system. Real-time RT-PCR using SYBR green master mix was performed. The oligonucleotide primer sequences are listed in Table 1. The data obtained by real-time RT-PCR were analyzed by the comparative threshold cycle (Ct) method^24, 51^. The relative expression of each gene compared to the expression level of β-actin was calculated, and was subsequently normalized according to the value for cells.

**Table 1:** Primer sequences used for myogenesis related mRNA expression in L6 skeletal muscle cell lines.

Genes	Forward primers [5-3]	Reverse primers [5-3]
MHC	5-GAAGGCCAAGAAGGCCATC-3	5-CTCGCCTCTCGTGTTTTCG-3
Myogenin	5-GCAGTGCCATCCAGTACATTGAGC-3	5-GGAAGGTGACAGACATATCCTCCAC-3
MyoD	5-GGCAAAGTGCTCTACAACGGCTA-3	5-CTGGAATCTCGTGCCTTCG-3
β-actin	5-TCACCGAGGCCCCTCTGAACCCTA-3	5-GGCAGTAATCTCCTTCTGCATCCT-3

### Intracellular nitric oxide, cGMP and NOS analysis

The intracellular NO radical was measured according to the method previously reported by our group^52^. We used the DAF-FM Diacetate probe to estimate the total NO inside the cell. Adherent L6 cells in the 6-well plate in DMEM media were exposed to an O_2_ flow of 200 SCCM for 0 and 3 min exposure. Each treated/untreated sample was transferred to a microcentrifuge tube. After washing three times with PBS, 20 mM DAF-FM Diacetate was added to each sample. The cells were incubated at 30 °C for 1 h and were then washed twice with PBS, then read at 495/515 (ex./em.) nm using a microplate reader. For the measurements of the cGMP levels and NOS activity, we used the cGMP & NOS immunoassay kit (abcam, USA) and (Bio Vision, USA) following the standard protocol provided with the kit.

### Western blot analysis

L6 cells were exposed to an O_2_ flow of 200 SCCM for 3 min upto on 8 days incubation. After exposure, protein extraction of the cells was performed. The whole cell protein extracts from treated/untreated cells were lysed in a RIPA buffer (Cell Signaling Technology, USA) and the extracted proteins were subjected to electrophoresis in 12% SDS-PAGE and blotted onto nitrocellulose membranes. The membrane was probed with the MHC, Erks, and AMPK antibodies. MHC was used for protein expression, while Erks and AMPK were used for phosphorylation analysis (Cell Signaling Technology, USA). The bands were detected using the Super Signal West Pico Chemiluminescent substrate (Pierce, Rockford, IL, USA) and images was taken using a Vilver imaging system (Vilver, Upland, CA, USA).

### Statistical analysis

All values are represented as the mean ± S.D. of the indicated number of replicates. Statistical analyses of the data were performed using the Student’s t-test to establish the significance between data points, with significant differences based on P < 0.05 or P < 0.01.

## Electronic supplementary material

Supporting information
