# Supplementary material for: Influence of Nitric Oxide generated through microwave plasma on L6 skeletal muscle cell myogenesis via oxidative signaling pathways
Source: Sci Rep. 2017 Apr 3;7:542. doi: 10.1038/s41598-017-00154-3 (PMC5427886; doi:10.1038/s41598-017-00154-3)
Supplement: Supplementary file 1 — Supporting information [file 41598_2017_154_MOESM1_ESM.pdf]

Supporting information

Influence of Nitric Oxide generated through microwave plasma on L6 skeletal muscle cell differentiation via oxidative signaling pathways

Naresh Kumar[#], Priyanka Shaw[#], Han Sup Uhm*, Eun Ha Choi* and Pankaj Attri*

Plasma Bioscience Research Center/ Department of Electrical and Biological Physics, Kwangwoon University, 20 Kwangwon-Ro, Nowon-Gu, Seoul 139-701, Korea

Titles and legends to figures

Figure S1. Immunofluorescence actin, tublin, and nucleus images of L6 cells on 2nd days after exposure with 3 min of 200 sccm of oxygen (control without exposure is taken as 0 min)

Figure S2. Immunofluorescence actin, tublin, and nucleus images of L6 cells on 4th days after exposure with 3 min of 200 sccm of oxygen (control without exposure is taken as 0 min)

Figure S3. Immunofluorescence actin, tublin, and nucleus images of L6 cells on 8th days after exposure with 3 min of 200 sccm of oxygen (control without exposure is taken as 0 min)

Figure S4. NOS activates and gene expression analysis. (a) NOS activates after exposure with NO (200 of O_2 flow rate) for 3 min and in the presence of 50 µM of GSNO on day 2, day 4, and day 8, (b) Myogenic differentiation regulated mRNA expression such as myoD, MHC, and myogenin gene expression analysis after exposure with 50 µM GSNO on day 8. The relative value of mRNA expression of these genes was measured by real-time RT-PCR. β-actin was used as a reference gene. All values are expressed as ± SD in triplicate.

Figure S5. Western blot analysis of the MHC, Erks, APMK and Erks & AMPK phosphorylation on 8th day after exposure with NO (200 of O2 flow rate) for 3 min. β-tublin was used as the loading control.

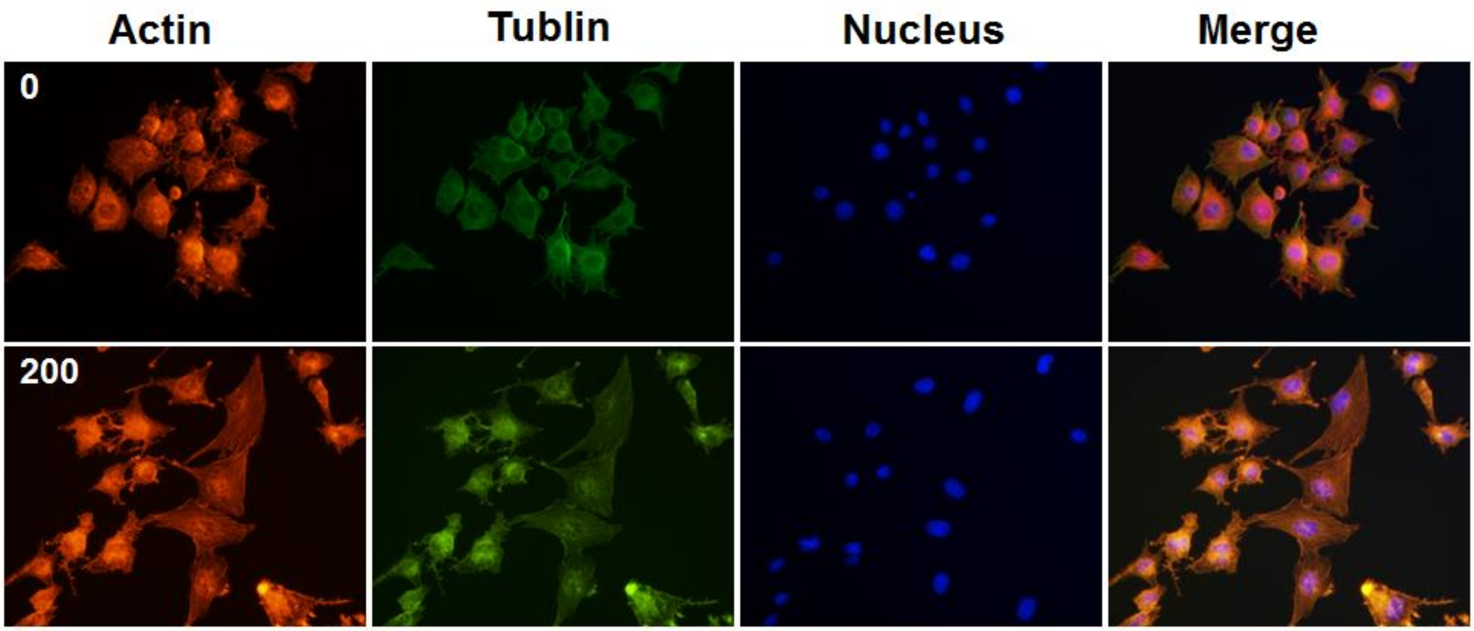

Figure S1

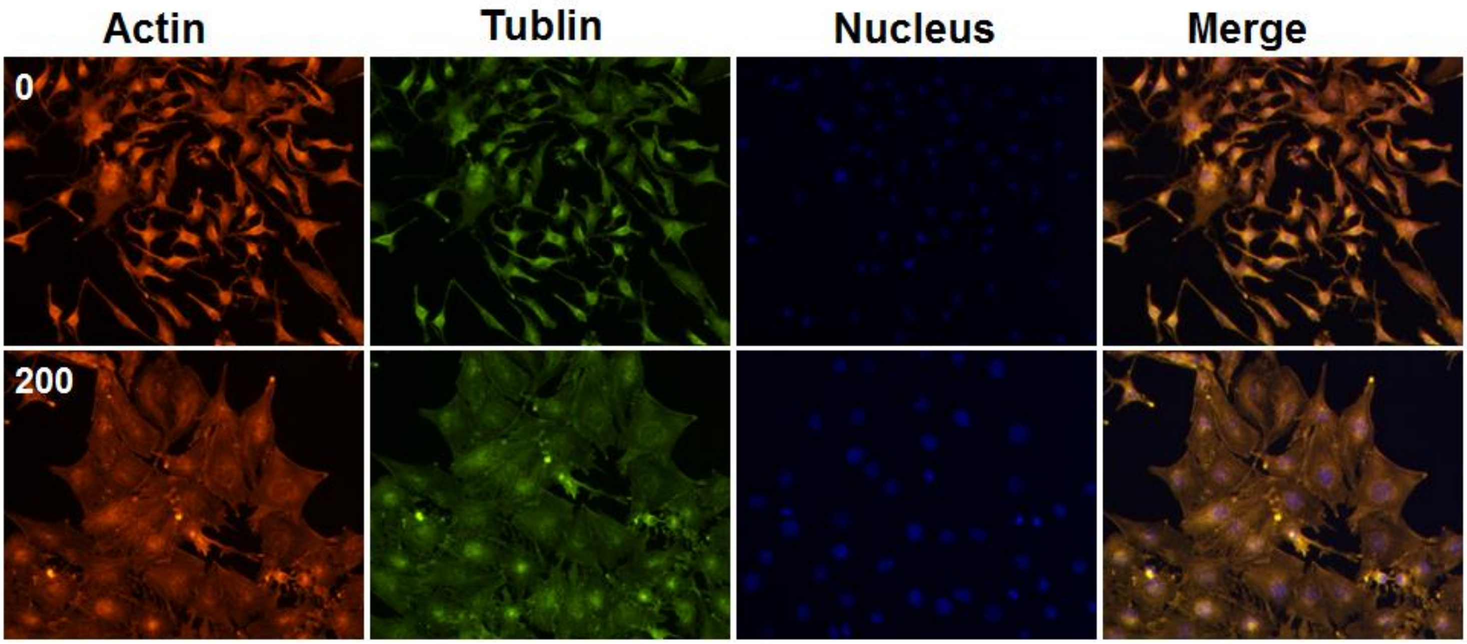

Figure S2

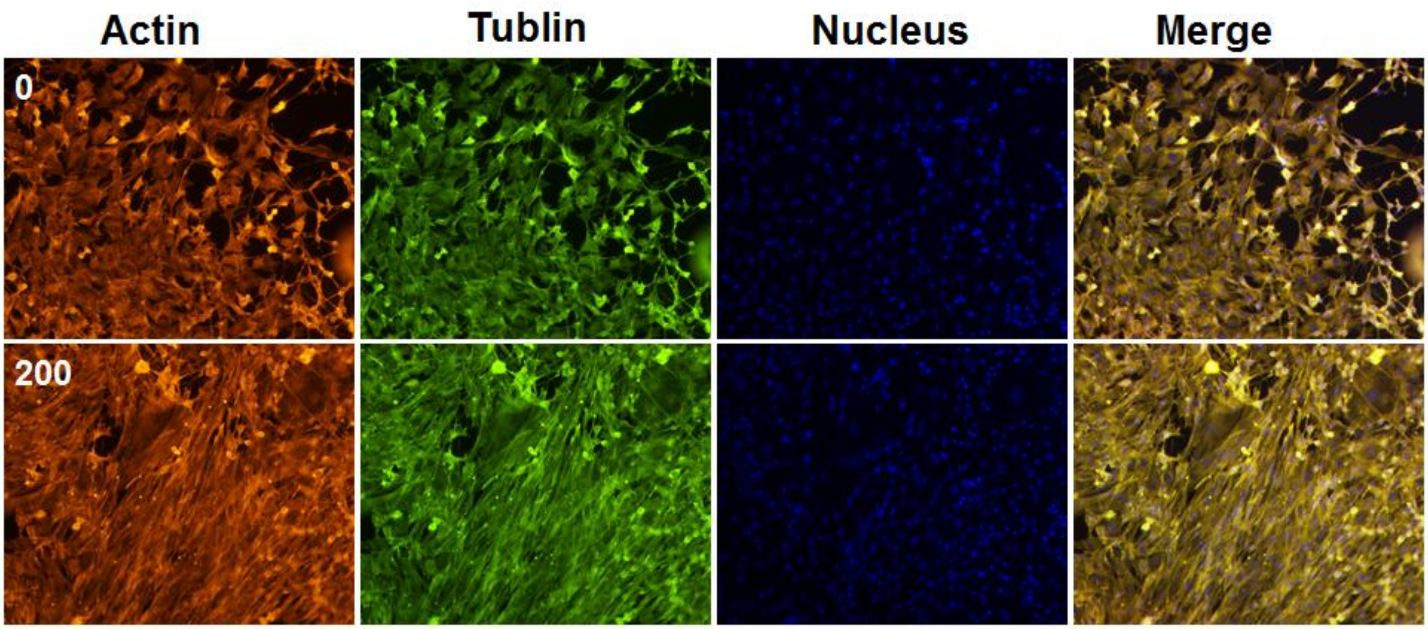

Figure S3

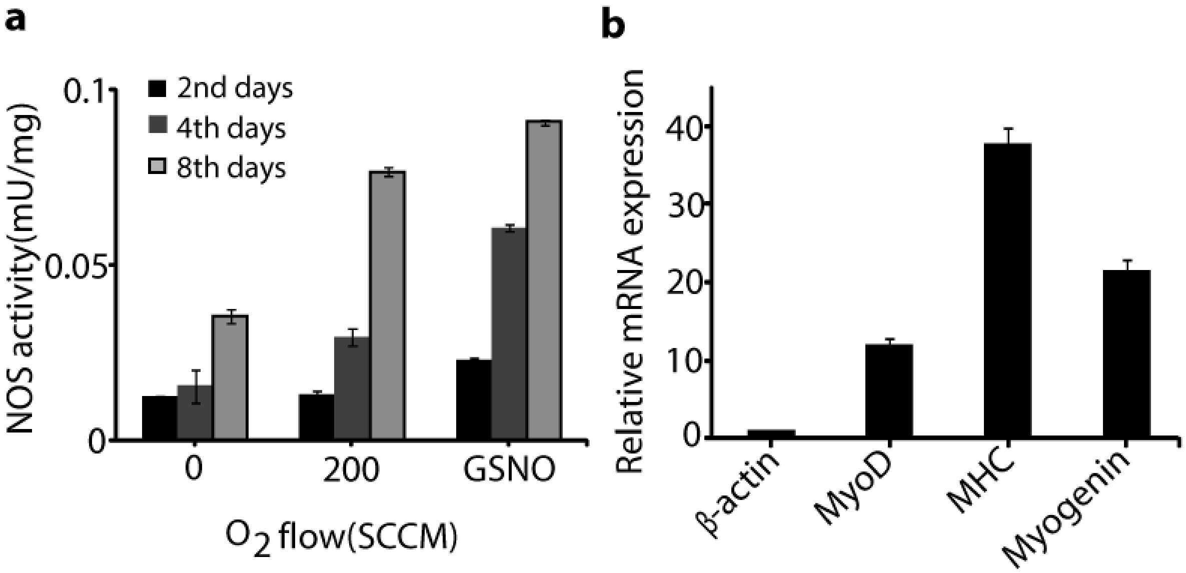

Figure S4

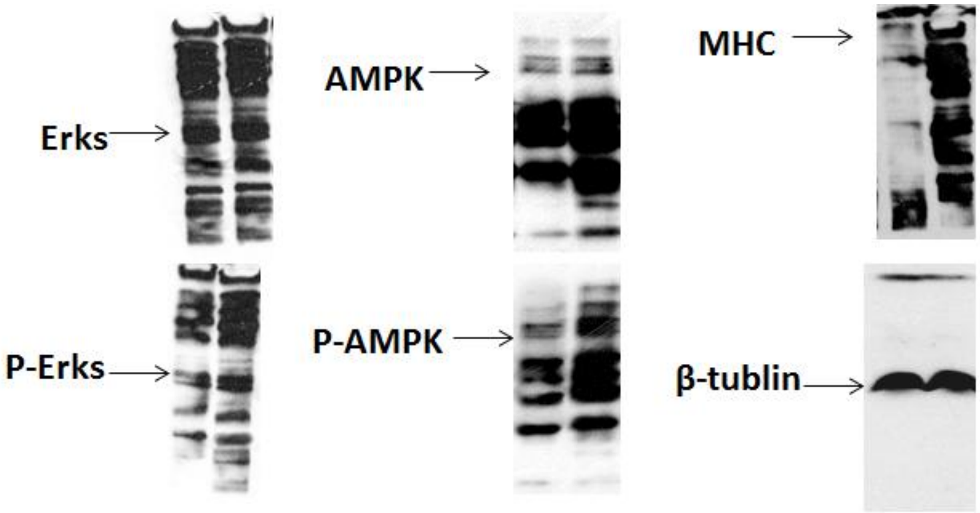

Figure S5
